# Why Do Parents Take Their Children to Informal Healthcare Providers? Insights from Bangladesh

**DOI:** 10.4269/ajtmh.25-0461

**Published:** 2026-02-12

**Authors:** Aparna Mangadu, Jane Putnam, Jyoti Bhushan Das, Sarah Dallas, Mohammad Saeed Munim, Ridwan Mostafa Shihab, Isthtiakul Islam Khan, Olivia Hanson, Zahid Hasan Khan, Md. Taufiqul Islam, Debashish Biswas, Mohammad Ashraful Amin, Firdausi Qadri, Daniel T. Leung, Ashraful Islam Khan, Melissa H. Watt

**Affiliations:** ^1^ Division of Infectious Diseases, Department of Internal Medicine, Spencer Fox Eccles School of Medicine at the University of Utah, Salt Lake City, Utah;; ^2^ Honors College, University of Utah, Salt Lake City, Utah;; ^3^ Health Systems and Population Studies Division, International Centre for Diarrhoeal Disease Research, Bangladesh, Dhaka, Bangladesh;; ^4^ Infectious Diseases Division, International Centre for Diarrhoeal Disease Research, Bangladesh, Dhaka, Bangladesh;; ^5^ Spencer Fox Eccles School of Medicine at the University of Utah, Salt Lake City, Utah;; ^6^ School of Population and Global Health, University of Western Australia, Perth, Western Australia, Australia;; ^7^ Department of Population Health Sciences, Spencer Fox Eccles School of Medicine at the University of Utah, Salt Lake City, Utah

## Abstract

In low- and middle-income countries, informal healthcare providers are often the first point of contact for pediatric care. The present qualitative study, conducted in Bangladesh, was designed to explore why parents seek pediatric care from informal providers. Through in-depth interviews with 12 parents of children under 5 years of age, key drivers for visiting informal providers were identified, including geographic and financial accessibility, as well as trust and familiarity. Parents expected informal providers to offer them informed and effective treatment but did not have a clear understanding of evidence-based treatment of pediatric diarrhea. Although informal providers may meet families’ immediate needs, they can also undermine formal healthcare visits and follow-ups that are essential to supporting children’s long-term health. The study findings highlight the need for targeted health education to improve parents’ knowledge, awareness, and informed decision-making. Strengthening trust in formal providers and addressing accessibility barriers is essential to improving pediatric health outcomes in low-resource settings.

## INTRODUCTION

In many low- and middle-income countries (LMICs), access to formal healthcare services remains limited.^1^ In these environments, informal healthcare providers, including drug vendors, traditional healers, and untrained allopathic providers, play a central role in delivering healthcare services, offering medical advice and treatment, and meeting other healthcare needs for the population, especially the rural poor.^2^

Although informal providers bridge critical gaps in healthcare access, they do not replace the value of regular and consistent engagement with formally trained healthcare practitioners, who are equipped to provide evidence-based prevention and treatment of acute and chronic ailments.^3^ This is especially true for pediatric populations, in which early prevention is key and both short- and long-term impacts of diseases can be life-threatening and often require longitudinal management.^4^^,^^5^

In Bangladesh, informal providers known as “village doctors” practice allopathic medicine largely without formal medical training and play an important role in providing healthcare services, consultations, and medications.^6^ In many parts of the country, village doctors are the first and most accessible point of care for the rural population. However, their clinical decisions are frequently influenced by external pressures, including financial incentives, the need to retain clientele, and the demands and expectations of patients and their families.^7^

Because village doctors operate in a competitive, profit-driven marketplace, their clinical decisions and behaviors are influenced by patient expectations. Recent studies have revealed that external pressure from patients and their families can lead to inappropriate treatment practices by informal providers, including unnecessary antibiotic use^8^^,^^9^ and delays in referring patients to higher level healthcare facilities.^10^ Additionally, families of pediatric patients often lack awareness of the importance of formal pediatric healthcare, resulting in poor follow-up even when referrals are provided.^10^

Given the critical role and well-established benefits of regular contact with formal practitioners in improving pediatric health outcomes,^11^ there is an urgent need to understand the motivations of parents and other caregivers who take their child to informal providers. At the same time, formal healthcare systems also face challenges, including accessibility barriers and financial incentives, that may influence pediatric care decisions. The aim for the present study is to qualitatively explore the motivations and expectations of parents when taking their child to a village doctor in Bangladesh, offering insights into the types of care that matter most to families and how parents make decisions about their child’s health while navigating the complex healthcare landscape shaped by formal and informal systems.

## MATERIALS AND METHODS

The present study was undertaken collaboratively by members of the research team at the International Centre for Diarrheal Disease Research, Bangladesh (icddr,b) and the University of Utah. All data were collected in the Sitakunda Upazila (subdistrict) of the Chattogram District in southeastern Bangladesh. The Sitakunda Upazila covers ∼500 square kilometers and has a population of ∼383,000 people.^12^ Through a community-based mapping exercise, 411 village doctor practices were identified in the Sitakunda Upazila.^13^

In June and July 2023, eight village doctor practices were intentionally selected from the original 411; from these eight practices, 12 caregivers of pediatric patients were recruited. Individuals were eligible if they were an adult (≥18 years old), responsible for a child under 5 years of age, and had taken their child to the village doctor with the primary complaint of diarrhea. Recruitment continued until data saturation was reached, at which point additional interviews no longer provided new insights.

Individual in-depth interviews (IDIs) were conducted at the village doctor practices by a member of the qualitative research team at the icddr,b. These interviews were conducted in Bangla and ranged in duration from 20 to 30 minutes. A semi-structured interview guide (Supplemental Appendix) included open-ended questions and follow-up probes and covered topics such as motivation for seeking village doctor care, past experiences with village doctors, and expectations during visits with village doctors. All IDIs were audio-recorded, transcribed verbatim, and translated into English to facilitate coding and thematic analysis by the international collaborative team.

Analysis was conducted in NVivo (version 12; Lumivero, Denver, CO) in accordance with an applied thematic analysis framework.^14^ The analysis involved several stages: familiarization with the raw data, coding, analytic memo writing, identification of emerging themes, and interpretation of the data. A codebook was developed to identify emerging themes across two domains: 1) parents’ motivations for seeking village doctor care, and 2) parents’ expectations of village doctors. The coding output was used to write analytic memos, which enabled a comprehensive understanding of the data and the synthesis of themes.

## RESULTS

The 12 participants included nine mothers and three fathers. The average age of the parents was 29 years (range: 18–40 years). Household income ranged from 6,000 to 25,000 Bangladeshi takas (∼$50 to $200), and few parents had beyond an eighth-grade education.

### Parents’ motivations for seeking care from village doctor.

Three themes characterized parents’ motivations for seeking care for pediatric diarrhea from a village doctor: 1) geographic accessibility, 2) financial accessibility, and 3) trust and familiarity (Table 1). Participants expressed a preference for village doctors due to their proximity to their residences, especially with limited transportation options. Although some acknowledged that formal healthcare facilities may offer a higher quality of care, participants consistently reported that they only went to formal healthcare facilities if the initial treatment from the village doctor was ineffective. Village doctors were preferred for their community locations and ability to provide house calls, which eliminated transportation barriers. Village doctors were also seen as more affordable because they offered similar treatments at lower costs. Finally, participants noted that they had longstanding relationships with the village doctors in their community, which fostered feelings of trust and familiarity.

**Table 1:** Representative quotes within each domain

Theme	Representative Quotes
Parents’ motivations for seeking village doctor care	
Geographic accessibility	*“The truth is, distance matters. The hospitals are not too far from here, but the VD’s chamber is walking distance from here. … It feels inconvenient to go to the hospital as it takes almost one hour to reach.” (Father of a 3-year-old, living in the community for 4 years)*
	*“He comes if he is called. Once, my baby was sick at midnight, the doctor [VD] went to his home to close his pharmacy, he was called at that moment and the doctor came in time. Doctors who live in the farthest areas would not come.” (Mother of a 3.5-year-old)*
Financial accessibility	*“If [the village doctor] gives the same medicines then why should we go to a big doctor (MBBS)? The medicines of a doctor with 800TK fees and the medicine of [the village doctor] are the same. We visit other doctors as well if the situation becomes so critical.” (Mother of a 3.5-year-old)*
	*“we can’t afford a specialized doctor all the time. They charge more, 500-700tk, there are also transportation and medicine cost. But those are out of our capacity.” (Mother of a 16-month-old, living in the community for 14 years)*
Trust and familiarity	*“Now the point is that I have been seeing Dr A here for treatment since my birth. In addition to this, all of our family took treatment from him. People go to others too, but actually I like him so I go to him. Apart from this, the effectiveness of medicine also works here.” (Father of a 1.6-year-old, living in the community for 20 years)*
	*“And he is a familiar face in this village. All of the village inhabitants go to him for treatment. As he had been serving this area’s people for a long time, now people have faith that they will be well if they get treatment from him.” (Father of a 3-year-old, living in the community for 38 years)*
Parents’ expectations of care from village doctors	
Informed treatment decisions	*“The main thing is that if a doctor can diagnose the diseases, then he [VD] can be able [to] give them better treatment. So, in terms of understanding a patient’s problems, I think he is a good doctor. There are a lot of doctors who prescribe medicines without a proper diagnosis. As he treats better than them, we don’t need to go outside for further treatment.” (Father of a 3-year-old, living in the community for 4 years)*
	*“He [VD] gives medicine after seeing the patients. If the first medicines don’t work, then he changes the medicine.” (Mother of a 1.5-year-old, living in the community for 11 years)*
Quick and effective treatment	*“Her fever decreased, and the diarrhea stopped. Just one day has passed since the medicines were given. He [VD] gave us the medicine the other day.” (Father of a 3-year-old, living in the community for 38 years)*

### Parents’ expectations of care from village doctors.

Two themes characterized parents’ expectations during a visit to a village doctor: 1) informed treatment decisions, and 2) quick and effective treatment (Table 1). Participants said that they expected a village doctor to consider the patient’s medical history and symptoms when determining the cause of illness and deciding on treatment. Additionally, they expected to receive treatment that provides a timely resolution of symptoms with minimal side effects. Although some parents specifically stated that they expected antibiotics, the majority expressed a general expectation for effective medication, suggesting limited knowledge of specific medication types or other evidence-based treatment options for managing diarrhea. No participants mentioned expectations for receiving rehydration therapy or guidance on non-pharmaceutical approaches for symptom management.

## DISCUSSION

Consistent access to the formal healthcare system is essential to ensure positive and long-term health outcomes for children.^11^ This access is often limited in LMICs like Bangladesh, where informal practitioners provide the majority of health-related services, including care for pediatric patients in rural areas.^2^ Although informal providers fill a critical gap, their limited medical knowledge can lead to inappropriate clinical decisions, potentially compromising children’s health.^7^ The aim for the present study was to qualitatively explore parents’ motivations and expectations when seeking pediatric care from a village doctor and identify opportunities for interventions to improve pediatric health.

Geographic and financial accessibility were identified as primary factors driving parents to seek pediatric care from village doctors rather than formal healthcare facilities. This finding is consistent with the existing literature, which reveals that fewer than 50% of people in low-resource settings can reach a registered healthcare facility within 60 minutes of walking.^15^^,^^16^ Although some studies have indicated that patients may be willing to accept higher costs and travel times to access formal healthcare facilities,^17^ there is a preference for local informal providers because of geographic and financial constraints.^18^ The present study’s findings add to this body of evidence and reinforce the need for targeted interventions to make healthcare more accessible and reduce family burden in low-resource areas. Given the importance of geographic location and proximity, interventions could also involve integrating village doctors into the formal healthcare system through structured training strategies to ensure adherence to appropriate clinical guidelines for pediatric care and encouraging timely referrals to formal health care facilities.

Trust and familiarity emerged as a significant motivator for seeking care from a village doctor. The authors of a systematic review in which the role of informal providers was examined offered similar insights, noting that informal providers in LMICs have high rates of trust and respect within the community.^2^ Community members in LMICs appreciate providers who understand cultural norms and context-specific factors.^19^ Building trust in formal providers requires systems-level interventions to create a facility culture of patient-centered care.^20^

Although parents commonly expected quick and effective remedies for their child’s condition, there was a gap in their understanding and knowledge of what an appropriate treatment plan might entail. This presents an opportunity for health education interventions to improve caregivers’ understanding of available, evidence-based treatment options for pediatric care, thereby promoting shared decision-making between providers and parents.

It is important to consider some limitations associated with the present study. The sample of parents was small and limited to a specific area in Bangladesh. The interviews were conducted with parents in the context of questioning them about their motivations and expectations for care related to pediatric diarrhea, which may limit the generalizability of the study findings to other pediatric ailments. Conducting the interviews at village doctor practices may have increased social desirability bias in favor of village doctors.

## CONCLUSION

In conclusion, key barriers and expectations influencing pediatric care-seeking behavior in Bangladesh have been identified in the present study. These findings can inform targeted approaches to address barriers to formal healthcare and encourage caregivers of pediatric patients to make informed decisions about their children’s health. The study findings also highlight the need for broader health system reform that recognizes the roles of both informal and formal healthcare providers. Strengthening collaboration between these systems is essential to promote effective treatment options and enhance pediatric care in low-resource settings.

## Supplemental Materials

10.4269/ajtmh.25-0461 Supplemental Materials
