# Supplementary material for: Why Do Parents Take Their Children to Informal Healthcare Providers? Insights from Bangladesh
Source: Am J Trop Med Hyg. 2026 Feb 12;114(4):801–4. doi: 10.4269/ajtmh.25-0461 (PMC12904251; doi:10.4269/ajtmh.25-0461)
Supplement: Supplemental Materials [file tpmd250461.SD1.pdf]

Interview guide for patient caregivers

The purpose of our interview today is to get your feedback as a caregiver of a child who is presenting to a village doctor with a primary complaint of diarrhea. We are mainly interested in **understanding what you expect and desire when you take your child to see the village doctor**.

As a reminder, your conversation with me today is for research purposes only. Nothing you say today will be shared with your doctor or any member of your clinical team. We hope you will feel comfortable talking openly and honestly about your experiences as a parent or caregiver. If any of my questions are not clear, please let me know and I can repeat or reword the question.

Do you have any questions before we begin?

I. Introduction

First, I'd like to talk to you about the events leading up to coming to the village doctor for your child's diarrhea.

1. Please tell me about yourself, and how you are related to the child.
    a. What is your role in caring for the sick child?
    b. Who else in your family is involved in caring for the sick child? What are their roles?

2. Please tell me briefly about your child's illness. (Probe: How long? How often?)
    a. Severity: How many stools a day? Is it bloody?
    b. Any other symptoms? [dehydration level etc.]

3. <u>Prior</u> to coming to this village doctor, did you do anything else to try and treat your child's diarrhea? (Traditional healer, Homeopathy or any doctor)
    a. Any medication used
        i. Type of medication
        ii. Where they got the medication
        iii. Why did you chose this medication?
        iv. Whether medication was helpful
    b. Other treatment
        i. Oral rehydration
        ii. Home remedies

II. Reasons for choosing a village doctor

Now I'd like to talk to you about why you decided to come see this village doctor for your child's diarrhea.

1. In your household, in what circumstances do you use village doctors?
    a. For what type of ailments/diseases do you go to a village doctor?
    b. What else would make you go to a village doctor instead of a clinic or hospital? (probe: severity, time of day, cost, distance)
    c. Who in your household makes the decision for when you should visit a village doctor?

2. In what circumstances would you go to a clinic or hospital instead of a village doctor?
    a. For what type of ailments/diseases do you go to a clinic or hospital instead of a village doctor?
    b. Who in your household makes the decision to visit clinic or hospital rather than a village doctor?

3. For your child's diarrhea today, what made you decide to visit the village doctor, instead of a clinic or hospital? [Probe: accessibility, easy communication, less fees/cost]

4. What was your reason for visiting *this particular* village doctor? [Probe: accessibility, cost, trust]

III. Experience with village doctor

Now I'd like to talk to you about your visit with the village doctor for your child's diarrhea.

1. Please tell me about your visit to the village doctor, from the time you arrived to the time you left.
    a. How did you explain your child's condition to the village doctor?
    b. What questions did the village doctor ask you?
    c. What treatments did you receive? (medication, ORS, IV fluid, etc.)
    d. Did the village doctor tell you why he gave you the treatment, or how he arrived at the decision for the treatment?
    e. What other advice or guidance did the village doctor give you?
    f. Were you told to come back for a follow up visit? (Probe: when)

2. At this village doctor, were you given any medication to help your child's diarrhea?

    If YES

   a. Please tell me about these medications. (Probe to find out if these are antibiotics; ask to see prescription/medication/strip or bottle if possible) ***Write down name of medication/s to add to spreadsheet***
   b. What instructions did you receive from the village doctor? [Dose/duration]
   c. What were you told about the reasons for these medications?
   d. How did you feel about receiving medications?
      If NO
   e. What were you told about the reasons medications were not given? What advice did you get?
   f. How did you feel about not receiving medications?

3. For some families, it's very important for them to get medications (like antibiotic pills) for their child's diarrhea. How important is it for you to get antibiotic pills when your child has diarrhea?
   a. Why do you say that this is important / not important?
   b. What do you think that antibiotic pills do for your child?
   c. How would you feel if you didn't receive antibiotic pills from the village doctor?
   d. What suggestion do you get if the doctor does not give you antibiotic pills?

4. Did the village doctor advise you to go to a clinic or hospital for the treatment of your child?
   If YES
   a. Why did they advise you to go to a clinic or hospital?
   b. Where did they advise you to go?
   c. Do you expect that you will go to the clinic or hospital? Why or why not?

5. If you don't mind telling me, I'd like to know how much your visit to the village doctor cost.
   a. Detailed cost by items: consultation, medication, other treatments, other costs

      Write down details of costs to add to spreadsheet

   b. Do you think these costs are reasonable? (Why / why not)
   c. If a village doctor comes to visit your home then do you have to pay extra fees?
   d. How does the cost of seeing a village doctor compare to going to a clinic or hospital?
   e. Are you satisfied with the services you got today? Why or why not, please explain.

IV. Areas for improvement

Thank you for all of your comments. To conclude, I'd like to hear your ideas about how the services from a village doctor could be improved.

1. What would you like to be different when you visit a village doctor?

2. Is there anything that a village doctor could do to make you feel more confident in the treatment you receive?

I have reached this end of my questions. Do you have anything to add on this topic of village doctors?
